# Thioredoxin-interacting protein in diabetic retinal neurodegeneration: A novel potential therapeutic target for diabetic retinopathy

**DOI:** 10.3389/fnins.2022.957667

**Published:** 2022-08-09

**Authors:** Chengzhi Liu, Wenkang Dong, Zhengshuai Lv, Li Kong, Xiang Ren

**Affiliations:** ^1^The First Affiliated Hospital of Dalian Medical University, Dalian, China; ^2^Department of Histology and Embryology, College of Basic Medicine, Dalian Medical University, Dalian, China

**Keywords:** TXNIP (thioredoxin-interacting protein), diabetic retinal neurodegeneration, diabetic retinopathy, thioredoxin, TXNIP modulators

## Abstract

Diabetic retinopathy (DR) is a common complication of diabetes mellitus and has been considered a microvascular disease for a long time. However, recent evidence suggests that diabetic retinal neurodegeneration (DRN), which manifests as neuronal apoptosis, a decrease in optic nerve axons, and reactive gliosis, occurs prior to retinal microvascular alterations. Thioredoxin-interacting protein (TXNIP) is an endogenous inhibitor of thioredoxin (Trx), and it acts by inhibiting its reducing capacity, thereby promoting cellular oxidative stress. In addition, it participates in regulating multiple signaling pathways as a member of the α-arrestin family of proteins. Accumulating evidence suggests that TXNIP is upregulated in diabetes and plays a pivotal role in the pathophysiological process of DR. In this review, we summarized the role of TXNIP in DRN, aiming to provide evidence for DR treatment in the future.

## Introduction

Diabetic retinopathy (DR) is a leading complication of diabetes and affects millions of individuals globally (Cheung et al., 2010). Earlier studies have linked DR to microvascular disease. DR is classified and treated clinically according to its vascular features (Hammes et al., 2011), which include microaneurysms, dot blot bleeding, and hard exudates in non-proliferative DR (NPDR), and retinal neovascularization and vitreous hemorrhage in proliferative DR (PDR) (Wilkinson et al., 2003). The treatment of DR focuses on vascular phenotypes by injecting anti-vascular endothelial growth factor (VEGF), and the effects are limited (Antonetti et al., 2021).

Structural and functional abnormalities of the retina occur in the early stage of DR, such as a decrease in optic nerve axons and reactive gliosis, which is described as diabetic retinal neurodegeneration (DRN) (Lynch and Abràmoff, 2017). An increasing number of studies have shown that DRN plays a pivotal role in the pathogenesis of DR and occurs earlier and more insidiously than microvascular lesions (Jonsson et al., 2016; Kadłubowska et al., 2016). There is considerable evidence that DRN contributes to retinal microvascular abnormalities in the early stage, suggesting that DRN is the precursor to some extent (Rossino et al., 2019). Our previous study revealed that early control of DRN is highly effective in delaying the progression of DR (Ren et al., 2017).

Thioredoxin-interacting protein (TXNIP) was first refined from leukemia cells (HL-60) treated with 1,25-dihydroxyvitamin D3 (vitamin D3) and named vitamin D3-upregulated protein-1 (VDUP1) (Chen and DeLuca, 1994). It was later identified as a protein that binds to thioredoxin (Trx) and inhibits its redox function (Schulze et al., 2004). Hence, it is also known as thioredoxin-binding protein-2 (TBP-2). Human TXNIP is a universally expressed protein that contains 391 amino acid residues. The *TXNIP* gene is encoded on chromosome 1q21.1 and is highly conserved among different species (Ludwig et al., 2001). As an endogenous inhibitor of the Trx system, cys63 and cys247 in TXNIP can form a mixed disulfide bond with mercaptan at the Trx active site and promote oxidative stress by inhibiting Trx function (Lu and Holmgren, 2014). TXNIP belongs to the α-arrestin protein family, and these intermediate scaffold proteins play an essential role in several signaling pathways (Patwari et al., 2009). Therefore, TXNIP plays many physiological roles independent of Trx binding (Spindel et al., 2012). It was previously confirmed that the downregulation of TXNIP or the upregulation of Trx reduces the production of intracellular reactive oxygen species (ROS) and alleviates retinal cell apoptosis (Ren et al., 2018). However, these mechanisms are complicated and require further investigation. This article summarizes the role of TXNIP in the pathogenesis of DRN and proposes new potential therapies based on the existing literature.

## TXNIP in diabetes

Thioredoxin-interacting protein is upregulated in response to glucose and has been identified as the highest glucose-induced gene in gene expression microarrays (Shalev et al., 2002). The expression of TXNIP is increased in the retina (Singh, 2013), pancreas (Shalev et al., 2002), kidney (Han et al., 2018), heart (Shen et al., 2018), peripheral nerves (Gao et al., 2020), and many other organs in patients with diabetes.

Physiologically, TXNIP functions as a dynamic nutrient sensor and contributes substantially to the dynamic balance of glucose regulation. To mitigate excessive glucose influx into the cell, TXNIP creates a negative feedback loop to regulate glucose uptake by binding to the glucose transporter (GLUT) (Wu et al., 2013). Under energy stress or control by insulin, AMP-activated protein kinase (AMPK) and Akt can phosphorylate TXNIP and cause its degeneration respectively, thereby preventing TXNIP-mediated endocytosis of GLUT transporters and leading to an increase in glucose influx (Dykstra et al., 2021). However, under pathophysiological conditions, such as diabetes, TXNIP is continuously induced by many factors, such as high glucose levels, causes β-cell dysfunction, and breaks this feedback loop.

### Translational level

The promoter of *TXNIP* contains multiple transcription factor binding sites, such as carbohydrate response element (ChoRE) and antioxidant element (ARE) (Dong et al., 2016). The expression of the carbohydrate-responsive element-binding protein (ChREBP) and MondoA increases when the glucose levels are high. The signal is transmitted to the nucleus, binding to ChoRE to activate the *TXNIP* promoter and promoting *TXNIP* gene transcription (Poungvarin et al., 2012; Richards et al., 2017). ChREBP is predominantly expressed in beta cells, the liver, and adipose tissue, whereas MondoA is mainly expressed in the skeletal muscle and heart (Thielen and Shalev, 2018). Forkhead box O1 transcription factor (FoxO1) competitively binds to the ChoRE sequence associated with the inhibition of TXNIP transcription (Kibbe et al., 2013; Zhong et al., 2021). Nuclear factor E2-related factor 2 (Nrf2) inhibits the expression of TXNIP and prevents its induction by high glucose and MondoA by binding to ARE (He and Ma, 2012). It has been confirmed that sulforaphane (SF), an Nrf2 activator (Russo et al., 2018), can alleviate mouse photoreceptor-derived (661w) cell apoptosis by reducing the expression of TXNIP (Lv et al., 2020).

As a branch of glycolysis, hexosamine pathway (HBP) flux increases in a chronic high glucose environment. The transcription of the *TXNIP* gene is mediated by HBP to some extent, which involves the recruitment of p300 histone acetyltransferase at the TXNIP promoter (Perrone et al., 2010). Moreover, Li et al. (2018) identified that p38 mitogen-activated protein kinase (MAPK) can be activated by ROS and induces TXNIP expression in the retinas of diabetic rats treated with high glucose. Streptozotocin (STZ)-induced hyperglycemia directly stimulates TXNIP expression through increased intracellular calcium ion (Ca^2+^) levels in the retina (Patwari et al., 2006). Insulin and glucagon-like peptide 1 (GLP-1) reduce β-cell TXNIP expression (Chen et al., 2006; Shaked et al., 2009). TXNIP can inhibit insulin production directly or by destroying the islet β-cells (Xu et al., 2013; Thielen and Shalev, 2018), forming a vicious cycle of TXNIP increase. Moreover, topical administration of GLP-1 receptor agonists has been proven to prevent retinal neurodegeneration in animal models (Hernández et al., 2016).

**FIGURE 1:**
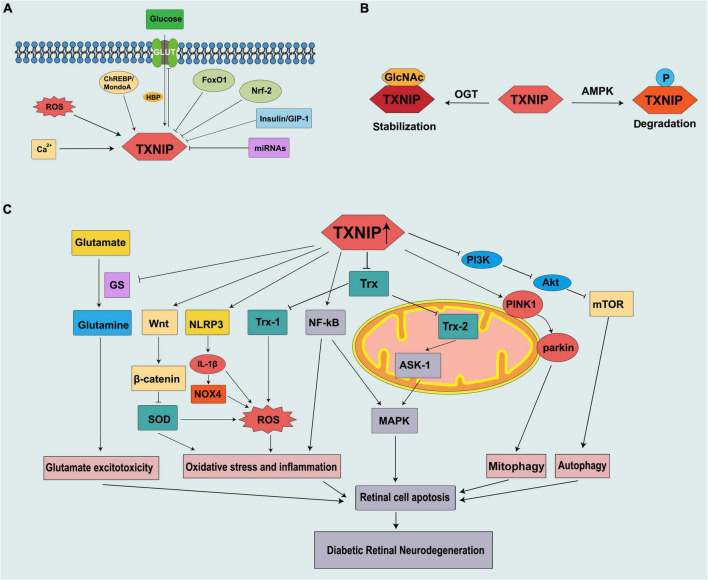
Schematic representation of thioredoxin-interacting protein (TXNIP) being regulated in diabetes and contributing to the pathogenesis of DRN. **(A)** Mechanism of TXNIP regulation at the translational level. **(B)** Mechanism of TXNIP regulation at the post-translational level. **(C)** TXNIP participates in different signaling pathways to promote DRN.

MicroRNAs (miRNAs) are small non-coding RNA molecules that participate in many vital biological processes by inhibiting messenger RNA (mRNAs) or by binding to the 3’untranslated region (3’UTR) of mRNA to limit protein synthesis (Krol et al., 2010). High glucose levels induce TXNIP upregulation, mediated by the downregulation of several miRNAs. Dong et al. (2016) demonstrated that microRNA-17 (miR-17) is downregulated by high glucose levels and stabilizes TXNIP. Moreover, Wang et al. (2020) showed that microRNA-20b-3p (miR-20b-3p), which inhibits inflammatory factors by inhibiting TXNIP to reduce pathological changes in the retina of DR rats, is expressed at low levels. Furthermore, high glucose levels induce the upregulation of circRNA_0084043, a type of circular RNA that mediates the downregulation of miR-128-3p in adult retinal pigment epithelial cells (ARPE-19) and causes TXNIP overexpression (Zhang et al., 2021; Figure 1A).

### Post-translational level

Thioredoxin-interacting protein can be modified by phosphorylation and O-GlcNAcylation. These two modifications are similar in many aspects and compete with each other. Thus, they are described as the “yin-yang” model (Hart et al., 1995, 2011). Energy stress leads to the phosphorylation of TXNIP by AMPK, resulting in its rapid degradation (Wu et al., 2013). As a nutrient sensor, like TXNIP, O-GlcNAcylation is also upregulated *via* HBP in the diabetic retina and high glucose conditions and is involved in the development of DR (Gurel and Sheibani, 2018). A recent study reported that the O-GlcNAcylation of TXNIP is increased in β cells, and an increased level of TXNIP is also observed in a diabetes model (Filhoulaud et al., 2019; Figure 1B).

## TXNIP in diabetic retinal neurodegeneration

Vascular abnormalities are the most evident clinical features of DR; however, evidence shows that retinal optic neuropathy occurs prior to vasculopathy. In db/db mice, the biological defect in the neural structure exists as early as the eighth week, which is characterized by a thinning of the outer nuclear layer (ONL) (Bogdanov et al., 2014). DRN results from neuronal apoptosis, reactive gliosis, glutamate excitotoxicity, and decreased neuroprotective factors (van Dijk et al., 2012; Chakravarthy and Devanathan, 2018). Excessive TXNIP production is observed in the retina that includes the retinal ganglion cells (RGCs) (Eissa et al., 2021), Müller cells (Devi et al., 2012), pericytes (PCs) (Devi et al., 2013), retinal pigment epithelium (RPEs) (Devi et al., 2019), and endothelial cells (ECs) (Perrone et al., 2009). However, it also has deleterious effects on these cells. TXNIP deficiency can reduce excessive autophagy and the ratio of B-cell lymphoma protein 2-associated X (Bax)/B-cell lymphoma protein 2 (Bcl-2) and inhibits retinal cell apoptosis (Yu et al., 2013). TXNIP ubiquitously exists in the cytoplasm and different organelles that include the nucleus and mitochondria, thereby affecting the function of various organelles and mediating multiple pathological processes.

### TXNIP regulates oxidative stress and inflammation in RDN

The retina is a unique organ that contains high concentrations of polyunsaturated fatty acids and requires high oxygen levels. Therefore, the retina and its vascular system are more susceptible to oxidative stress. Some evidence suggests that oxidative stress is the main cause of sterile retinal inflammation in diabetes (Rodríguez et al., 2019). Oxidative stress can contribute to and result from metabolic abnormalities induced by hyperglycemia in the retina.

Cellular redox balance (reduction/oxidation) is strongly regulated by the activities of many antioxidant systems, such as the Trx, glutathione, and glutaredoxin systems (Spindel et al., 2012). Trx1 and its reductase TrxR1 are expressed in the cytoplasm and nucleus, whereas Trx2/TrxR2 is a mitochondrial subtype (Lillig and Holmgren, 2007). During cell stress, TXNIP migrates to the mitochondria and releases apoptosis signal kinase 1 (ASK1) from Trx2 capture, which results in the release of cytochrome c from the mitochondria, cleavage of caspase-3, and apoptosis (Saxena et al., 2010). Many experimental studies have demonstrated an increase in ASK1 induced by TXNIP, which is associated with retinal neurodegeneration and diabetes (Sreekumar et al., 2009; Abdelsaid et al., 2014). TXNIP also induces the activation of nuclear factor-kappa B (NF-κB), which plays a pivotal role in inflammation and colocalizes with retinal Müller cells and ECs (Perrone et al., 2009; Al-Gayyar et al., 2011). NF-κB and ASK1 activate the apoptotic p38 MAPK/c-Jun N-terminal kinase (JNK) pathway and promote retinal cell death (Al-Gayyar et al., 2011).

Thioredoxin-interacting protein is a multifunctional and inducible protein with functions independent of its combination with Trx. The NOD-like receptor protein-3 (NLRP3) inflammasome, composed of NLRP3, adaptor protein apoptosis associated speck-like protein containing a CARD (ASC), and procaspase-1, is responsible for the production and secretion of interleukin (IL)-1β (Martinon et al., 2002). IL-1β can induce retinal mitochondrial dysfunction, mitochondrial DNA damage, and apoptosis (Gu et al., 2019). IL-1β reduces the promoter activity of *pri-miR-590* DNA to downregulate miR-590-3p, thereby targeting NLRP1 and activating the NADPH oxidase 4 (NOX4)-mediated pathway to promote retinal cell apoptosis. In addition, NOX4 further promotes TXNIP expression by inducing ROS (Gu et al., 2019). TXNIP plays a potential role in sterile inflammatory processes and innate immunity through the activation and releasing of IL-1β by the NLRP3 inflammasome in diabetes and oxidative stress. Furthermore, TXNIP can be induced by ROS, leading to NLRP3 activation in diabetes (Schroder et al., 2010; Lu et al., 2018). TXNIP can also activate the Wnt/β-catenin signal pathway (Zhang et al., 2021). The activation of the Wnt/β-catenin pathway downregulates the expression of antioxidant enzymes, such as superoxide dismutase (SOD)1 and SOD2, which further exacerbates cellular oxidative stress (Liu et al., 2019).

### TXNIP regulates autophagy/mitophagy in RDN

Autophagy is the primary physiological process by which cells eliminate damaged organelles and defective proteins and is considered an important regulator of homeostasis (Levine and Kroemer, 2019). However, under extreme pressure, it can lead to programmed cell death (Maiuri et al., 2007). Autophagy plays a “double-edged sword” role in DR (Gong et al., 2021). The unc-51 like autophagy activating kinase 1 (ULK1) complex participates in the initiation of autophagy, and the mammalian target of rapamycin (mTOR) functions as the negative regulator of autophagy by phosphating ULK1 (Jung et al., 2009). Excessive autophagic flux is induced by retinal neuronal cells under high glucose exposure (Rosa et al., 2016; Adornetto et al., 2021). Müller cells are located from the outer layer to the inner layer of the retina. They are responsible for the communication between retinal blood vessels and neurons and provide nutrition for the retinal nerve cells. TXNIP promotes autophagy by inhibiting the PI3K/Akt/mTOR pathway in Müller cells, which leads to Müller cell damage and disease progression. A decrease in the fluorescence intensity of LC3B was observed in the retina after the knockout of TXNIP in STZ-induced diabetic rats, indicating that autophagy was downregulated (Ao et al., 2021). In addition, we observed the overexpression of Trx, which shows the inhibitory activity of TXNIP, ameliorated photoreceptor cell degeneration in DR *via* AMPK-mediated autophagy, and exosome secretion (Ren et al., 2022).

Mitophagy is a specialized process that removes damaged, obsolete, and dysfunctional mitochondria through lysosomal degradation. It is a cytoprotective mechanism that ensures cell survival and tissue protection (Levine and Kroemer, 2019). However, excessive mitophagy flux may lead to the damage to mitochondria, which continue to produce ROS while producing less adenosine triphosphate (ATP), leading to oxidative stress and retinal apoptosis, which eventually develops DRN (Singh et al., 2017). PINK1 accumulates on the outer membrane of depolarized mitochondria, recruits E3 ubiquitin ligase parkin, mediates ubiquitination of mitochondrial membrane proteins, and activates mitophagy (Skeie et al., 2021). TXNIP and ROS cause mitochondrial damage and fragmentation through dynamin-related protein (Drp1)-mediated mitochondrial (MT) fission and PINK1/parkin-mediated mitophagy in Müller cells and TXNIP knockout prevents these events (Devi et al., 2017). Similar results are seen for RPEs (Devi et al., 2019).

### TXNIP regulates glutamate excitotoxicity in RDN

Glutamate is the most abundant and important amino acid in the nervous system. It plays an important role in synaptic transmission and maintaining the normal function of nerve cells. Glutamate metabolism is very important in maintaining a healthy retina and its dysregulation can lead to the development of DR. Moreover, under pathological conditions, such as diabetes, the accumulation of glutamate in the retina causes the overexcitation of N-methyl-D-aspartate receptors (NMDARs), which causes calcium influx and eventually leads to DRN (Yu et al., 2008). Glutamine synthetase (GS) benefits glutamate clearance through metabolizing glutamate to glutamine. Moreover, glutamate is an excitatory neurotransmitter, and it can accumulate in the retina to toxic levels due to diabetes and thus cause excitotoxicity damage to the retinal neurons (Rolev et al., 2021). Another mechanism of how glutamate excitotoxicity mediates neurodegeneration in the retina is partly through nitric oxide (NO), which causes mitochondria fragmentation in RGCs, leading to NMDA receptor upregulation and enhanced oxidative stress, thus triggering neurodegeneration (Nguyen et al., 2011). Zhou et al. (2016) reported that TXNIP suppresses the expression of GS by IL-1β, which leads to Müller cell dysfunction and further induces ganglion cell apoptosis (Figure 1C).

## TXNIP: as novel potential therapeutic target for diabetic retinopathy

Substantial evidence suggests that hyperglycemia promotes the expression of TXNIP through multiple pathways. An increased TXNIP level has emerged as a novel key factor in DRN (Figure 1), and the inhibition of TXNIP expression *in vivo* and *in vitro* effectively prevents early pathology of DR (Perrone et al., 2010; Ren et al., 2018). We summarized the potential moderators targeting TXNIP to treat DRN by blocking the expression and downstream pathways of TXNIP (Table 1).

**TABLE 1:** List of drugs/compounds that have been identified to alleviate diabetic retinal neurodegeneration (DRN) in experimental models.

Drug/compound	Target	DRN model	Intervention	Observed effect	References
microRNA-20b-3p	TXNIP	The DR rat models were established by intraperitoneal injection with 0.1 mol/L sodium citrate buffer solution (pH 4.6) containing STZ (60 mg/kg) in male rats aging 8–10 weeks	Rats were injected with miR-20b-3p mimics at the vitreous cavity 1 week after modeled	The up-regulation of miR20b-3p could repress TXNIP in retinal tissues of DR rats, thereby decreasing the expression of NLRP3, Caspase-1, IL-1β, IL-18, and TNF-α	Wang et al., 2020
1,25-dihydroxy vitamin D3 (vitamin D3)	ROS/TXNIP/NLRP3	The DR rat models were established by intraperitoneal injection with STZ (60 mg/kg) in 8-week-old male Sprague-Dawley rats	Rats were injected with calcitriol at a dose of 233.3 U/kg body weight/week dissolved in tea oil for 6 months, starting 4 weeks after modeled	Vitamin D3 exerted its protective effect by decreasing the level of ROS production, thus down-regulating TXNIP expression and blocking the activation of NLRP3, and inhibiting Retinal Cell Apoptosis in diabetic rats	Lu et al., 2018
Verapamil	Ca2+ channel/TXNIP	The DR rat models were established by a single rapid injection of STZ (50 mg/g) intravenously in the tail vein in Wistar rats	Rats were treated with verapamil (10 mg/kg, oral) for 4 months, after 48 h post-STZ insult	The treatment with verapamil enhanced Trx-R activity and significantly inhibited TLR4, TXNIP, and NLRP3 inflammatory bodies, thereby preventing the progression of diabetic retinopathy	Eissa et al., 2021
Luteolin	TXNIP	The DR rat models were established by the administration of STZ (60 mg/kg) in Wistar rats (200-250 g)	Rats were treated with luteolin (50 mg/kg, oral) for 4 months after the induction of DR	Luteolin reduced the expression of TXNIP, NOX4, and oxidative stress	Yang et al., 2021
Metformin	ChREBP/TXNIP	The DR mouse models were established by intraperitoneal injection with STZ in male C57BL/6 mice	Mice were treated with metformin (25 mg/kg, oral) for 8 weeks, after the final STZ injection	Metformin alleviated GCL cell death by inhibiting the O-GlcNAc modification of ChREBP, consequently decreasing TXNIP levels in the diabetic retina	Kim et al., 2017
Sulforaphane (SF)	AMPK/TXNIP	The mouse photoreceptor-derived (661w) cell line was treated with different concentrations of AEGs	661w cells were treated with SF (0.1 or 0.5 μM) for 3 h in 5% CO2 at 37°C	SF delayed diabetes-induced retinal photoreceptor cell degeneration by inhibiting ER stress, inflammatory reactions, and the expression of TXNIP through the activation of the AMPK pathway	Lv et al., 2020
A triple-drug combination (TXNIP-IN1, Mito-Tempo, and ML-SA1)	TXNIP	The rat retinal Müller cell line (rMC1) was treated with high glucose (25 mM)	rMC1 cells were treated in combination with the three drugs and were maintained in high glucose for 3 days in DMEM/F12 medium	Targeting TXNIP and mitochondrial–lysosomal stress are effective in normalizing mitophagic flux and transcription factor TFEB and PGC-1α nuclear translocation to maintain the mitochondria–lysosome axis and autophagy process in rMC1	Singh and Devi, 2021
Gambogic acid (GA)	Nrf-2/TXNIP	The PREs (ARPE-19 cells) were treated with high glucose (30 mM)	ARPE-19 cells were treated with 200 μM GA (0.5, 1, 2, 5, 10, and 20 μM) for 24 h	GA can dose-dependently down-regulate the expression of NLRP3 inflammasome components, including TXNIP	Chen et al., 2021

Given the beneficial effects of TXNIP deficiency, gene therapy using vectors, such as lentivirus and adeno-associated viral vectors, to transfer genes to the retina may be another choice. Nucleic acid constructs containing a proximal TXNIP promoter linked to a redox gene or shRNA that reduces oxidative stress and inflammation may be used to treat DR (Lalit et al., 2018). Clustered regularly interspaced short palindromic repeats (CRISPR)-associated 9 (CRISPR/cas9) an emerging technology, is used for genome editing in animals and cultured cells. Recent reports showed its progress in clinical trials of some genetic diseases (Arnold, 2021). Devi et al. (2017) applied the CRISPR/cas9 method to knockout TXNIP and observed that it can prevent high glucose-induced Müller cell line rMC1 mitochondrial damage and mitochondrial autophagy.

Thioredoxin-interacting protein is notably upregulated and is not restricted to specific cell types in the diabetic retina; therefore, intraocular administration is theoretically feasible. Nevertheless, the dose needs to be controlled within the physiological level and varies from person to person. A report also shows the adverse effects of excessive downregulation of TXNIP. In a study on age-related macular degeneration (AMD), researchers observed that sustained oxidative stress mediates TXNIP downregulation and disrupts RPE cell function, thereby aggravating AMD (Ji Cho et al., 2019).

The inhibition of TXNIP promotes endogenous cell mass and insulin production, thereby becoming an attractive new candidate drug target for diabetes (Shalev, 2014; Wondafrash et al., 2020). Promising results have been obtained in experimental models of diabetes with anti-TXNIP agents, such as *S-Equol* (Chen et al., 2020), SRI-37330 (Thielen et al., 2020), MiR-17-5p (Liu et al., 2021), and fluoromethyl ketone (FMK) (Han et al., 2019). Moreover, TXNIP also participates in microangiopathy in DR, such as neovascularization (Duan et al., 2018), vascular permeability, and inner blood-retinal barrier (iBRB) breakdown (Perrone et al., 2009). Extensive literature is available regarding promising drugs that prevent retinal microvascular dysfunction induced by diabetes, directed at TXNIP. Minocycline and fenofibrate have demonstrated a protective effect on retinal vascular permeability by reducing ROS-induced TXNIP expression in diabetes (Chen et al., 2017; Li et al., 2018). Tang et al. (2021) revealed that melatonin, an ancient antioxidant, could maintain the iBRB integrity by upregulating the expression of tight junction proteins *via* inhibiting the p38MAPK/TXNIP/NF-κB pathway, thus decreasing the production of inflammatory factors to reduce retinal damage. Given the similar pharmacological effects of these drugs on inhibiting TXNIP expression, repurposing them for DRN treatment may be a direction for future research. Since DRN precedes microangiopathy in DR, revealing the mechanism of DRN is of great significance for the early screening and intervention of DR. We have attempted to review the evidence from experimental models that support the vital role of TXNIP in the etiology of DRN and potential treatment targeting TXNIP. Therefore, there is an urgent need to develop and evaluate the efficacy of these therapies in clinical trials. In summary, TXNIP played a vital in DRN and it could be a novel potential therapeutic target for DR.

## Author contributions

CL and XR conceived of this review topic. WD and CL retrieved and analyzed relevant documents. CL prepared the illustrations and wrote the manuscript. XR revised the manuscript. ZL, LK, and XR provided the funding support. All authors listed have made a substantial, direct, and intellectual contribution to the work, and approved it for publication.

## Abbreviations

DR: Diabetic retinopathy
DRN: diabetic retinal neurodegeneration
TXNIP: Thioredoxin-interacting protein
Trx: thioredoxin
NPDR: non-proliferative DR
PDR: proliferative DR
VEGF: anti-vascular endothelial growth factor
HL-60: Leukemia cell-60
vitamin D3: 1,25-dihydroxyvitamin D3
VDUP1: vitamin D3-upregulated protein-1
TBP-2: thioredoxin-binding protein-2
ROS: reactive oxygen species
ChoRE: carbohydrate response element
ARE: antioxidant element
ChREBP: carbohydrate-responsive element-binding protein
FoxO1: Forkhead box O1 transcription factor
Nrf2: Nuclear factor E2-related factor 2
SF: sulforaphane
HBP: hexosamine pathway
MAPK: mitogen-activated protein kinase
Ca2+: calcium ion
miRNAs: MicroRNAs
3'UTR: 3'untranslated region
MmiR-17: icroRNA-17
miR-20b-3p: MicroRNA-20b-3p
ARPE-19: adult retinal pigment epithelial cells
AMPK: AMP-activated protein kinase
ONL: outer nuclear layer
RGC: retinal ganglion cell
PC: pericytes
RPE: retinal pigment epithelium
EC: endothelial cell
ASK1: apoptosis signal kinase 1
NLRP3: NOD-like receptor protein-3
ASC: apoptosis associated speck-like protein containing a CARD
IL: interleukin
NOX4: NADPH oxidase 4
mTOR: mammalian target of rapamycin
NMDAR: N-methyl-D-aspartate receptor
GS: Glutamine synthetase
NO: nitric oxide
CRISPR/cas9: clustered regularly interspaced short palindromic repeats/CRISPR-associated 9
AMD: age-related macular degeneration
AFMK: fluoromethyl ketone
iBRB: inner blood-retinal barrier.
